# Diagnostic criteria of tuberculous sarcoidosis

**DOI:** 10.4103/0970-2113.53232

**Published:** 2009

**Authors:** J. R. Shah, Jagruti Hede, R. S. Mathur

**Affiliations:** *Department of Chest Diseases, Jaslok Hospital and Research Centre, Mumbai, India*

**Keywords:** Tuberculous sarcoidosis, tuberculosis, sarcoidosis

## Abstract

With the object to strengthen the clinical status of tuberculous sarcoidosis, we present in this article, the case records published in internationally recognized journals by specialists. From review of clinical material, we have also formulated a table that defines diagnostic criteria of tuberculous sarcoidosis.

## INTRODUCTION

The recognition of *M. tuberculosis* or its degraded products in some forms as one of the etiological agents of sarcoidosis is widely accepted and is even included in the recent textbooks of respiratory medicine and clinics in chest medicine.[1,2] With high prevalence of tuberculosis in India, and the increasing incidence of sarcoidosis, clinicians are expected to see many cases of tuberculous sarcoidosis. Scadding, who gave the definition of sarcoidosis that was followed for half a century also coined the term ‘tuberculous sarcoidosis’ in 1962 to distinguish clinically defined tuberculosis and sarcoidosis from the patients who showed combined clinico-pathologic features of both. Most clinicians in respiratory practice have come across such a situation as reported in recent correspondence of ‘Lung India’; Dixit reported three such cases after reading the article tuberculous sarcoidosis.[3]

In order to improve the diagnostic criteria of tuberculous sarcoidosis, we went through the case histories of patients published in recognized journals. The published material comprised 39 case reports, 34 by Scadding;[4] three by Noppen;[5] one by Wong;[6] and one by Bedi.[7] Out of the published material we present here the case histories, which indisputably quantify the criteria of tuberculous sarcoidosis.

We describe here some selected case reports published in the literature.

## CASE 1: CONCOMITANT TUBERCULOSIS AND SARCOIDOSIS

A 35-year-old Chinese woman initially presented with histologically and bacteriologically confirmed tuberculous lymphadenitis. She was also found to have thrombocytopenia, elevated serum alkaline phosphatase, and bilateral lung infiltrates.[6] After 15 months of antitubercular treatment, despite the resolution of cervical lymphadenopathy, she started to experience dyspnea. Also, chest radiograph appearance, thrombocyte count, and liver biochemistry, all had deteriorated. Histologic findings from tissues obtained via transbronchial biopsy and open lung biopsy were consistent with sarcoidosis but also showed the presence of mycobacterial DNA by the polymerase chain reaction. She subsequently achieved a very good response clinically, radiographically, hematologically, and biochemically with one year of corticosteroid treatment for her sarcoidosis, and she remained relapse-free afterwards.

## CASE 2: PLEURAL EFFUSION IN SARCOIDOIS

A 48 year-old-female, housewife, presented with moderate right-sided lymphocytic exudative pleural effusion.[7] Rest of her lung fields were normal and there was no hilar lymphadenopathy on chest skiagram. She was managed with R, Z, H, E, and corticosteroids and became normal in two months when Z and E were stopped. However, after four months of therapy, she developed cough and breathlessness and chest skiagram; and CT scan showed bilateral hilar and mediastinal lymphadenopathy (without ring enhancement or caseation), and bilateral parenchymal infiltration. Her serum ACE level was 150 IU/ml (Normal 17-70 IU/L) and tuberculin test was negative with 5TU of PPD. Her transbronchial lung biopsy revealed noncaseating granulomas consistent with the diagnosis of sarcoidosis.

## CASE 3 AND 4: THE RELATION BETWEEN SARCOIDOSIS AND TUBERCULOSIS

### Case 3

A 22 year old man had tuberculous adenitis in the left side of his neck; this softened and was aspirated. The aspirated pus showed *tubercle bacilli*. Four years later, he developed a generalized enlargement of lymph-nodes, affecting the cervical, axillary, and inguinal group of lymph nodes; a node removed for biopsy showed noncaseating epithelioid-cell tubercles. The spleen was easily palpable. After one year, he developed iridocyclitis in the right eye. A chest radiograph showed bilateral hilar lymph node enlargement, with diffuse fine mottling mainly in the middle zones of both lungs. A tuberculin test gave a moderate reaction to 100 t.u. Over the next six months, all these manifestations gradually subsided, the lymph nodes and the spleen were no longer palpable, the eye was free from inflammatory changes, and the chest radiograph was clear. He remained quite well, when a lymph node swelling appeared again in the left side of the neck. The skin now reacted to 10 t.u. with an area of induration 20 × 20 mm. He was treated with isoniazid and p-aminosalicylic acid (P.A.S.). The lymph node softened and sterile pus was aspirated from it; after this the adenitis subsided completely, and the patient has remained well since then.[4]

### Case 4

An 18 year old woman was observed at a chest clinic for four years as a contact of her husband, who had been found to be suffering from pulmonary tuberculosis. The chest radiograph of the woman remained clear, but their son born during this time was found to have skin sensitivity to tuberculin at the age of one year. The woman was first seen at the Brompton Hospital in January 1956, because a small area of faint mottling had been found in the upper zone of the right lung in a chest radiograph. Sputum examination at this time produced tubercle bacilli on culture. In May 1956, she was admitted to hospital. Her skin reacted to 10 t.u. Tubercle bacilli was again found on culture and this time from a gastric lavage specimen. Treatment with isoniazid and P.A.S. was started, but the lung shadows slowly and steadily spread. She was readmitted in May 1958; the skin now failed to react even to 1000 t.u. Liver biopsy showed noncaseating tubercles. Prednisolone was added to the antitubercular drugs in June 1958, which resulted in rapid clearing of the radiographic shadows. However, after the prednisolone was gradually withdrawn and stopped in July 1959, she started to feel tired; and in October 1959, a radiograph showed some recurrence of the abnormal shadows.[4]

In this case the sequence of events is striking; there was a steady and uninterrupted progression from exposure to tubercular infection, through the development of a localized lung lesion with tuberculin sensitivity and tubercle bacilli in the sputum, to a state typical of sarcoidosis, clinically, histologically, and in response to treatment.

## CASE 5: ALL THAT CASEATE IS NOT TUBERCULOSIS

A 48 years old female, presented with right facial palsy, bilateral parotid swelling, predominantly dry cough and dyspnoea of one year duration.[8] She also complained of dysphagia, nasal regurgitation, decreased vision in both the eyes since 4-5 years. There was no history of exposure to organic or inorganic dust or history suggestive of collagen vascular disease. She had been evaluated for these symptoms and in view of parotid swelling cytology suggestive of tuberculous inflammation, was started on antituberculosis drugs, but without any relief of symptoms. She also had right-sided VII, IX, X, XI cranial nerve palsy. Hematological investigations were normal. X-ray chest showed bilateral ground glass opacities. Sputum was negative for acid-fast bacilli. Sputum culture did not grow any organism. Parotid swelling cytology showed epitheloid cell granuloma with caseous necrosis. Ophthalmologic examination for fundus was normal. Ultrasonography of abdomen showed splenomegaly. Tuberculin skin test was negative. Computed tomography (CT) thorax showed interlobular and intralobar septal thickening involving bilateral lung fields with mediastinal lymphadenopathy, which showed homogenous enhancement following contrast injection. Magnetic Resonance Imaging (MRI) brain performed in view of cranial nerve palsy showed cerebral and cerebellar atrophy but no focal abnormality. Angiotensin Converting Enzyme (ACE) levels were 145 U/(elevated) and gallium scan showed focal accumulation of gallium in right hilar region with no other abnormal focal uptake elsewhere in the body which was typical of sarcoidosis, confirming the diagnosis of sarcoidosis. Antitubercular therapy was stopped and oral corticosteroid therapy initiated with improvement in symptoms. In this case, although the diagnosis of tuberculosis was made initially based on cytology showing granuloma with caseation necrosis, the long duration of illness, its response to steroids, negative sputum cultures excluded an infectious etiology. However, multisystemic sarcoidosis could be diagnosed in view of homogenously enhancing mediastinal lymphadenopathy on CT scan thorax, tuberculin skin test negativity, elevated ACE levels, and nerve palsies.

## CASE 6: SARCOIDOSIS, TUBERCULOSIS OR BOTH?

Mark Noppen reported that patients presenting features of both sarcoidosis and tuberculosis (whether or not in causative relationship), can be puzzling to the chest physicians.[9] In the European Journal of Internal Medicine he described case histories of three patients with “overlap features” of both disorders, and in whom extensive diagnostic workup (though not including polymerase chain reaction search for mycobacterial DNA), and response to therapy did not allow unequivocal classification into diagnosis of either sarcoidosis or tuberculosis.[5]

We analyzed clinical manifestations of the above published material along with over 20 cases of sarcoidosis which we have seen at this hospital,[10,11] and were categorized into tuberculous sarcoidosis. We formulated the following Table to establish as far as can be precise the clinical criteria that would distinguish tuberculous sarcoidosis from tuberculosis and sarcoidosis.

## DIAGNOSTIC CRITERIA FOR DIAGNOSIS OF TUBERCULOUS SARCOIDOSIS

Age: 25-45 years

Gender: No gender predisposition

History of previous tuberculosis infection which was adequately treated with antitubercular therapy with adequate drug combinations, dosages and duration.

History of close contact with patient having tuberculosis infection or family history of tuberculosis or association with Type II diabetes mellitus.

Onset: Asymtomatic or with mild fever, anorexia and loss of weight.

Important symptoms: Chronic dry nonproductive cough, breathlessness on exertion.

Important signs: Involvement of multiple nodes, for example, scalene or cervical groups of lymph nodes, at multiple locations.

Bilateral bibasilar end inspiratory velcrow crackles on auscultation of chest.

## INVESTIGATIONS

Raised inflammatory markers: ESR

Increased SACE level.

Tuberculin test: Positive or negative.

At times patient may develop localized granulomatous reaction at the site of tuberculin test.

Sputum: Positive or negative for *M. tuberculosis*.

Culture: Negative for *M. tuberculosis*.

PCR of biopsy tissues is positive for *M. tuberculosis*.

Biopsy of involved site shows noncaseating granulomas with classical confluence at times showing caseation necrosis.

X-ray Chest: Bilateral symmetrical hilar and right paratracheal lymphadenopathy.

Pulmonary parenchymal infilteration, which is usually bilaterally symmetrical involving the mid-zone and upper-zone and absence of cavitations.

CT scan of chest: Micro nodular and macro nodules which have characteristic distribution in peri-bronchovascular region, subpleural interstitium and interlobular septa.

No expected response to antitubercular therapy. Dramatic response to steroids, with resolution of symptoms.

The list, however, should be considered open for modification with increasing observations on patients of tuberculous sarcoidosis.

## CONCLUSION

With the object to strengthen the clinical status of tuberculous sarcoidosis, we present in this article, case records published in internationally recognized journals by specialists. From review of clinical material, we have also formulated diagnostic criteria of tuberculous sarcoidosis.
